# Effect of implant position on clinical outcomes of two implant overdentures: a 3-year randomized clinical trial

**DOI:** 10.1186/s12903-026-08441-0

**Published:** 2026-05-09

**Authors:** Mohammed Hussein Raafat, Khloud Ezzat Mourad, Moustafa Abdou Elsyad

**Affiliations:** 1 Department of Prosthodontics, Faculty of Dentistry, Mansoura University, Mansoura, Egypt; 2 Department of Prosthodontics, Faculty of Dentistry, Mansoura National University, Gamasa, Egypt

**Keywords:** Implant overdenture, Crestal bone loss, Retention, Patient satisfaction

## Abstract

**Background:**

Reviewing the literature, the optimal implant position for 2-implant retained mandibular overdentures, which would be associated with improved peri-implant tissues, higher retention values, and better patient satisfaction, remains uncertain. The study was designed to assess the effect of the implant position of two implant-retained mandibular overdentures on clinical outcomes (peri-implant soft tissue health, bone loss, retention, and patient satisfaction).

**Materials and methods:**

Ninety edentulous patients with unsatisfactory retention of their mandibular conventional dentures were included in this study and received 2-implant mandibular overdentures with Locator attachments. The patients were randomly divided into three groups based on the positions of the implants. Group LA (implants in lateral incisor positions), Group CA (implants in the canine positions), and Group PM (implants in the premolar positions). Changes in the peri-implant soft tissue health (plaque, gingival scores, and probing depth) and the retention values of overdentures were evaluated at prosthesis insertion (T0), 6 months (T6), and 12 months (T12) after insertion. The peri-implant marginal bone loss changes were evaluated one year (T12), 2 years (T24), and three years after insertion (T36). A visual analog scale (VAS) assessed patient satisfaction for conventional dentures (CD), LA, CA, and PM at T6.

**Results:**

At T6 and T12, the highest plaque and gingival scores were noted with the PM, followed by LA, and the lowest scores were observed with CA. For all observations, the highest pocket depth was noted with PM, followed by CA, and the lowest pocket depth was noted with LA. CA and PM showed significantly higher bone loss compared to LA. The highest retention forces were exhibited by PM, followed by CA, and the lowest retention values were noted with LA. Regarding retention, stability, occlusion of the mandibular prosthesis, and ease of chewing, the PM group had the greatest patient satisfaction, then CA and LA. The lowest satisfaction scores were noted with CD.

**Conclusions:**

In selecting the implant position for two-implant mandibular overdentures, it is crucial to balance several key clinical outcomes. The chosen location for the implants should effectively prioritize bone preservation, the health of peri-implant soft tissues, retention, and overall patient satisfaction. Positioning the implants near the lateral incisors is advantageous for minimizing bone loss, while placement near the canines supports optimal soft tissue health. Conversely, positioning them near the premolars maximizes retention and enhances patient satisfaction.

**Clinical trial registry number:**

(NCT06166316) (12/13/2023).

## Background

Edentulism is commonly associated with significant functional and psychosocial challenges, particularly concerning mandibular dentures. Although many patients can adapt to complete dentures, maxillary prostheses generally offer a more satisfactory experience. In contrast, mandibular dentures present greater difficulties related to retention and stability [1, 2]. Traditionally, before the emergence of endosseous implants, conventional complete dentures remained the primary treatment for edentulous patients for over a century. However, the limitations of mandibular dentures frequently resulted in inadequate stability and retention, leading to a substantial number of patients expressing dissatisfaction with their prosthetic solutions, even when designed to the highest clinical standards [3]. Fortunately, the introduction of implant-supported overdentures has effectively addressed many of these issues, providing a more stable and reliable option for individuals struggling with mandibular dentures [4]. 

Based on extensive literature reviews [5, 6], the consensus proposals from McGill and York recommend that 2 implants should be considered the minimum treatment for a completely edentulous mandible, taking into account retention, stability, masticatory performance, patient satisfaction, cost, and clinical time. Several resilient attachments that can permit vertical and hinge movements have been used for such overdentures. These attachments include balls and sockets with spacers, resilient telescopes, magnets, and locators [7, 8]. Locators are highly favored attachments due to their minimal thickness and self-aligning capabilities, which can correct up to 40 degrees of implant angulation [9]. Their low profile and ability to accommodate implant angulation make them a preferred choice in many clinical scenarios [10, 11]. Locators provide enhanced stability and retention while simplifying hygiene maintenance. Research indicates that locators can improve Oral Health-Related Quality of Life (OHRQoL) scores compared to ball attachments [12]. It has been shown in the literature that the outcome of the mandibular 2 implant overdenture is not affected by the type or design of the implant or attachment systems or soft tissue indices, but rather the over-time maintenance requirements of these overdentures [13–16]. 

Meta-analyses of randomized controlled trials have shown that there are only slight differences in the risk of implant loss and average peri-implant bone loss between the use of 2 or 4 implants [5]. For two-implant overdentures, it remains unclear where the implants should be placed in the mandible to create the most favorable biomechanical conditions and ensure long-term success. The shape and size of the jawbone, as well as the remaining bone volume in the canine area, may influence where implants are placed. This could mean putting the implants slightly more to the front for the lateral incisors or further back for the premolars. Research has shown that the position of the implant has a bigger impact than its angle or size [17, 18]. Numerous studies indicate that implants ought to be positioned in the interforaminal region, particularly at the canine location, to achieve optimal outcomes [19, 20]. However, the clinician may need to consider lateral incisor location when there is a lack of an adequate amount of bone at the canine position. Furthermore, biomechanical factors may require the placement of implants in a more distal location (at the premolar area) to reduce stress on the peri-implant bone during rotational denture movements [19, 21]. 

Currently, clinical research lacks sufficient evidence to definitively ascertain the impact of various implant locations on stress distribution around implants. However, certain studies propose that the insertion of implants in lateral incisor areas results in the least amount of stress, making this placement the preferred alternative [22–24]. Conversely, other studies suggest that lower stress levels are evident with implants positioned at the first premolar site, rather than the lateral incisor and canine sites [19, 25]. Monitoring peri-implant soft tissue health and marginal bone loss can be considered a good indicator of overloading or excessive stress on the implants [26]. Assessing prosthetic treatment also requires a focus on patient-centered outcomes, which can be measured effectively using oral health-related quality of life (OHRQoL) [27] and visual analog scales (VAS).

Loss of retention in overdenture attachments is influenced by multiple factors, including wear and fatigue of the attachment materials. These can be caused by various biomechanical and clinical factors, such as differing stress levels applied to these attachments, implant angulation, and maintenance practices [28–30]. Moreover, it has been found in previous studies that different implant positions for implant-overdenture can lead to varying retention levels, potentially impacting patient satisfaction outcomes [31–33]. This study aimed to examine the effect of implant location (lateral incisors, canines, and premolar regions) on peri-implant tissue, retention, and patient satisfaction with two-implant overdentures. The null hypotheses of the study propose that there would be no significant impact of different implant positions on peri-implant soft tissue health, retention values, and patient satisfaction.

## Methods

### Participant selection and study design

This three-arm randomized controlled clinical trial was conducted on ninety edentulous patients (50 males and 40 females, aged between 43 and 82 years, with a mean age of 64 years) complaining about stability and retention problems of their mandibular complete dentures were chosen from the Prosthodontic Department’s outpatient clinic. The criteria for inclusion were as follows: adequate bone quantity (Cawood and Howell class IV-VI [34]) and adequate bone quality (Lekholm and Zarb classes I-III [35]) in the anterior areas of the mandibular jaw to accommodate dental implants (minimum 11 mm length and 3.7 mm diameter). Patients with systemic diseases impacting bone metabolism, as well as those with smoking, alcohol, and parafunctional habits, were not included in the research.

A sample size of 30 individuals per group (with 15% expected to drop out) was determined to offer 90% power with an alpha level of 0.05 and an effect size of.4 mm (SD = 0.2 mm) in marginal bone loss between groups based on the findings of a prior study [36]. All participants received thorough explanations before giving their written consent. The research protocol was registered at www.clinicaltrials.gov (NCT06166316) and approved by the Faculty of Dentistry’s Research Ethics Committee (A01011023RP). The study was conducted in adherence to the CONSORT guidelines governing clinical trials.

**Fig. 1:**
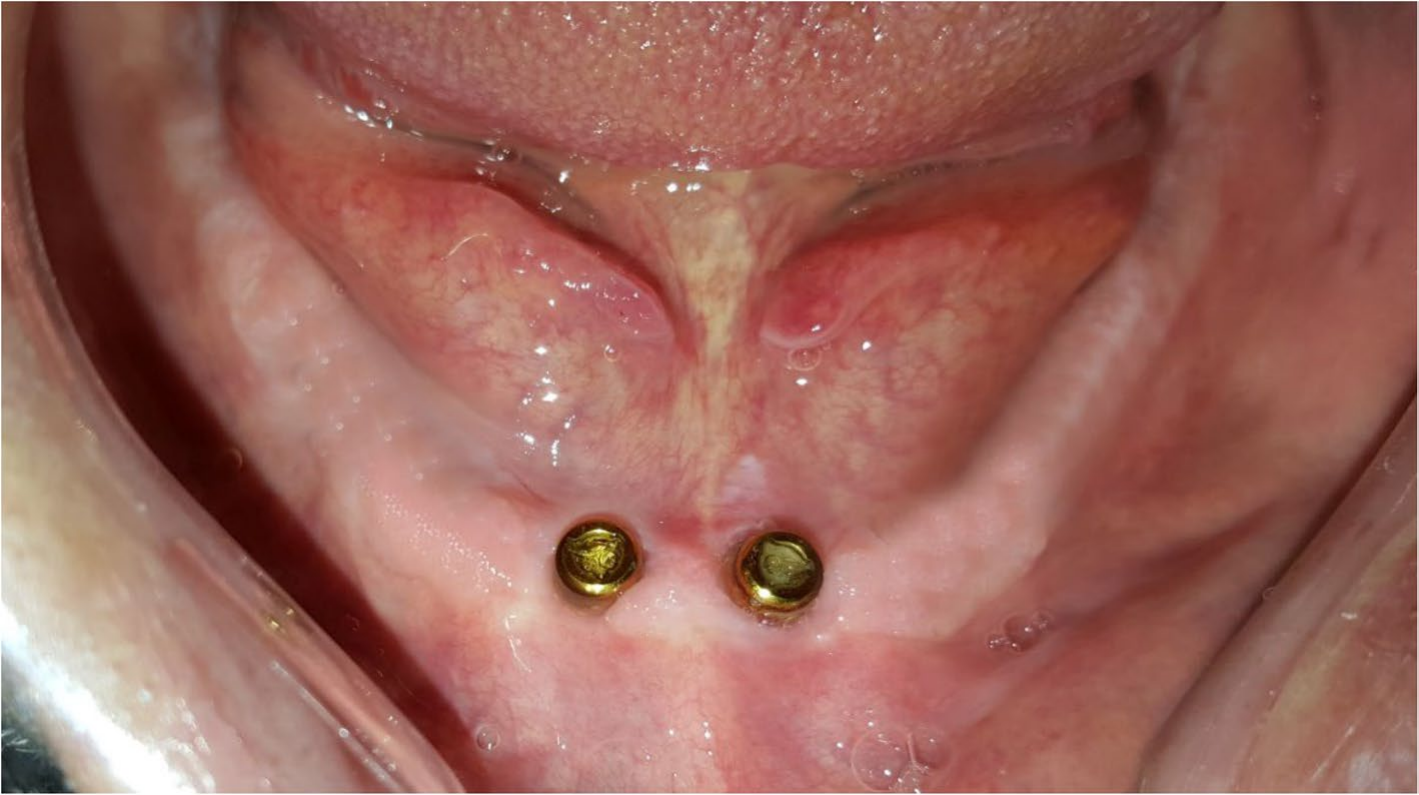
Group LA (implants in the lateral incisors’ position)

**Fig. 2:**
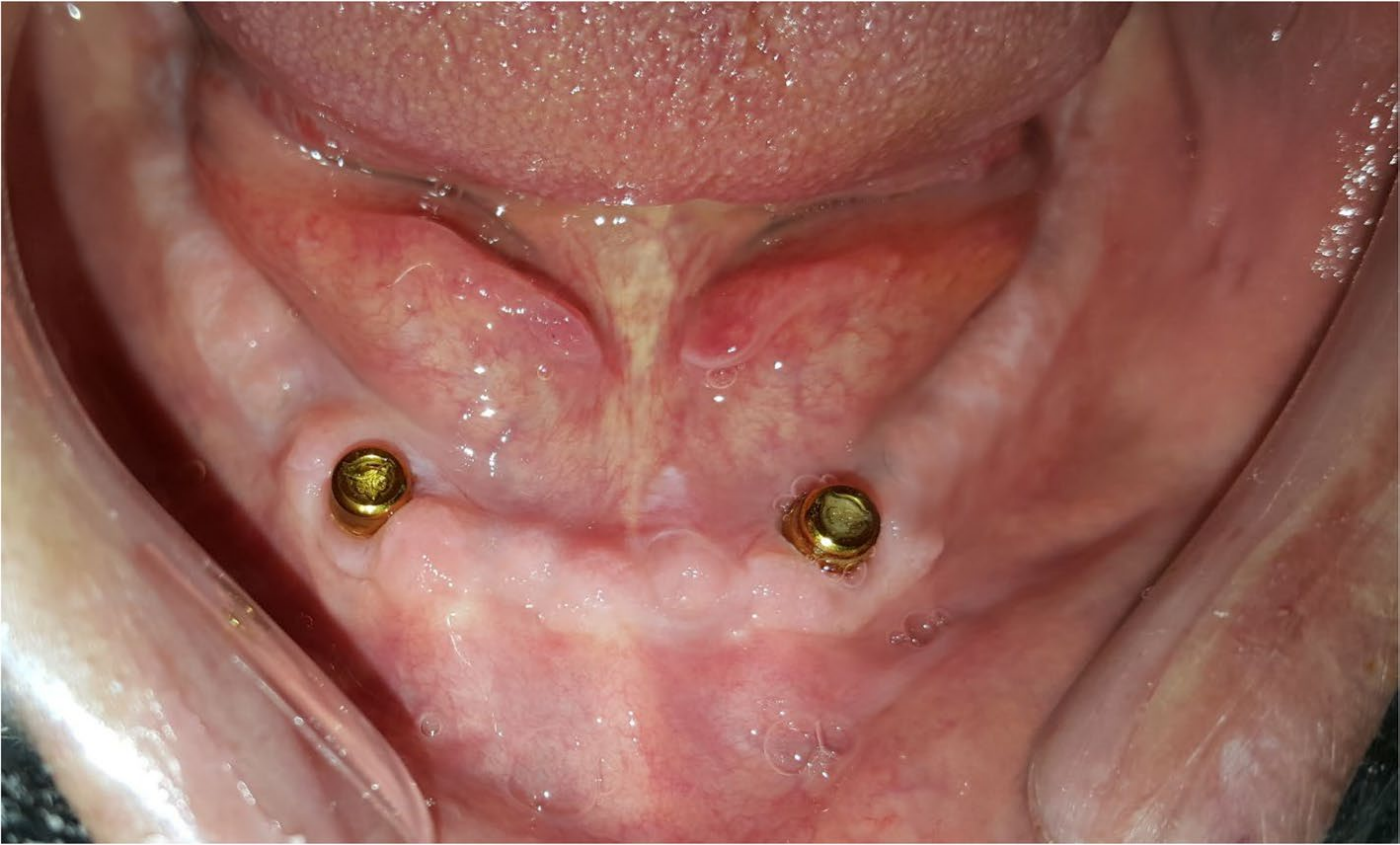
Group CA (implants in the canines’ position)

**Fig. 3:**
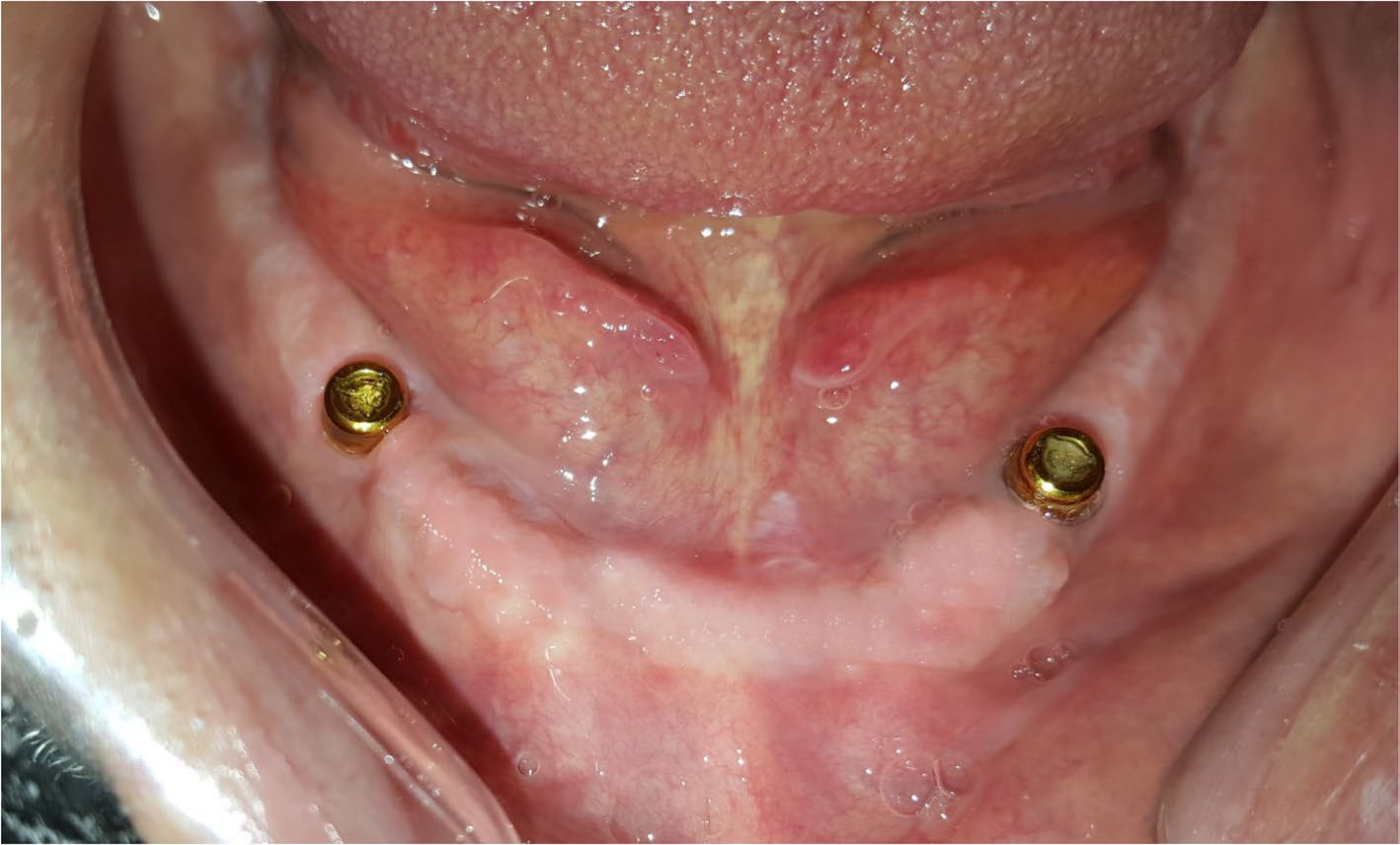
Group PM (implants in the premolars’ position)

Participants were stratified according to Gender, age, years of edentulism, number of previous dentures, and mandibular ridge height (baseline criteria). Random allocation of patients to groups was executed by a computerized balanced randomization method [37] to ensure that the groups were comparable before treatment (at baseline). An independent blind dental staff performed randomization and allocation of participants to the following groups: group LA, who received two implants in the lateral incisors position (Fig. 1), group CA, who received two implants in the canine position (Fig. 2), and group PM, who received two implants in the premolar position (Fig. 3). Locator attachments were used to attach the overdentures to the implants for all patients. Baseline characteristics of the participants are listed in Table 1.

**Table 1:** Baseline characters for groups

	Lateral group	Canine group	Premolar group	*P* value
Age (years)X ± SD	43–68(62.45 ± 2.4)	e50- 67(61.9 ± 3.7)	47–82(69.8 ± 2.9)	1
Gender (male/female) Frequency	7 M/5F	8 M/8F	5 M/7F	0.981
Bone height in (mm) X ± SD	12.3–14 (11.7 ± 1.15)	11.5–15 (12.7 ± 2.03)	10.9–13.7 (12.05 ± 1.19)	0.902
Period of edentulism (years) X ± SD	4–7 (5.01 ± 1.3)	5–8 (6.5 ± 1.45)	3–6 (4.2 ± 1.7)	0.6224

### Surgical and prosthetic procedures

For all participants, new dentures were fabricated with semi-anatomic acrylic teeth arranged according to the balanced occlusion concept [38]. Participants were instructed to use their dentures for 2 months before implant placement to improve adaptation. A duplicate mandibular denture was created as a radiographic guide, and each participant underwent cone beam computed tomography (CBCT) for pre-operative implant planning. Consequently, surgical guides were constructed by attaching metal tubes over the proposed implant positions. Two implants (TioLogic Dentaurum) were inserted, with a minimum torque of 35 Ncm, into the anterior mandible by the same oral and maxillofacial surgeon, using the conventional submerged surgical approach. If the minimum insertion torque was not achieved, an implant with a larger diameter was used if the bone width was adequate, or the implant was retained for osseointegration. Subsequently, the dentures were relieved over the implants and relined with a soft liner. Participants were instructed to adhere to a soft diet and to perform regular oral hygiene procedures. After 3 months, healing abutments were screwed to the implants for 2 weeks, then Locator abutments with suitable gingival height (Dentium, Co. Ltd.) were securely screwed into the implants and torqued to 30 Ncm. The mandibular dentures were sufficiently relieved over the locator abutments. The metal housings with associated attachment inserts (pink insert, medium retention values) were attached to the mandibular dentures by direct pick-up technique using cold-cure acrylic resin while the patients closed into centric occlusion. Changes were made to the occlusion as needed to achieve bilateral harmonious occlusal contact in centric and eccentric movements. In addition, all participants received detailed guidance on maintaining good oral hygiene, and follow-up appointments were scheduled for them.

### Clinical assessment of peri-implant soft tissue health

The parameters for clinical assessment of peri-implant tissue are: plaque index (PI), gingival index (GI), and pocket depth (PD). Measurements were done at the time of implant overdenture insertion (T0), after 6 months (T6), and after 12 months (T12). Modified gingival and plaque indexes were recorded according to Mombelli et al. [39]. Using a calibrated and plastic periodontal probe (Kerr, Rastatt), the distance between the border of the gingival margin and the probe tip was measured in mm and considered as the pocket depth (PD) [40]. 

### Marginal bone loss (MBL) measurements

**Fig. 4:**
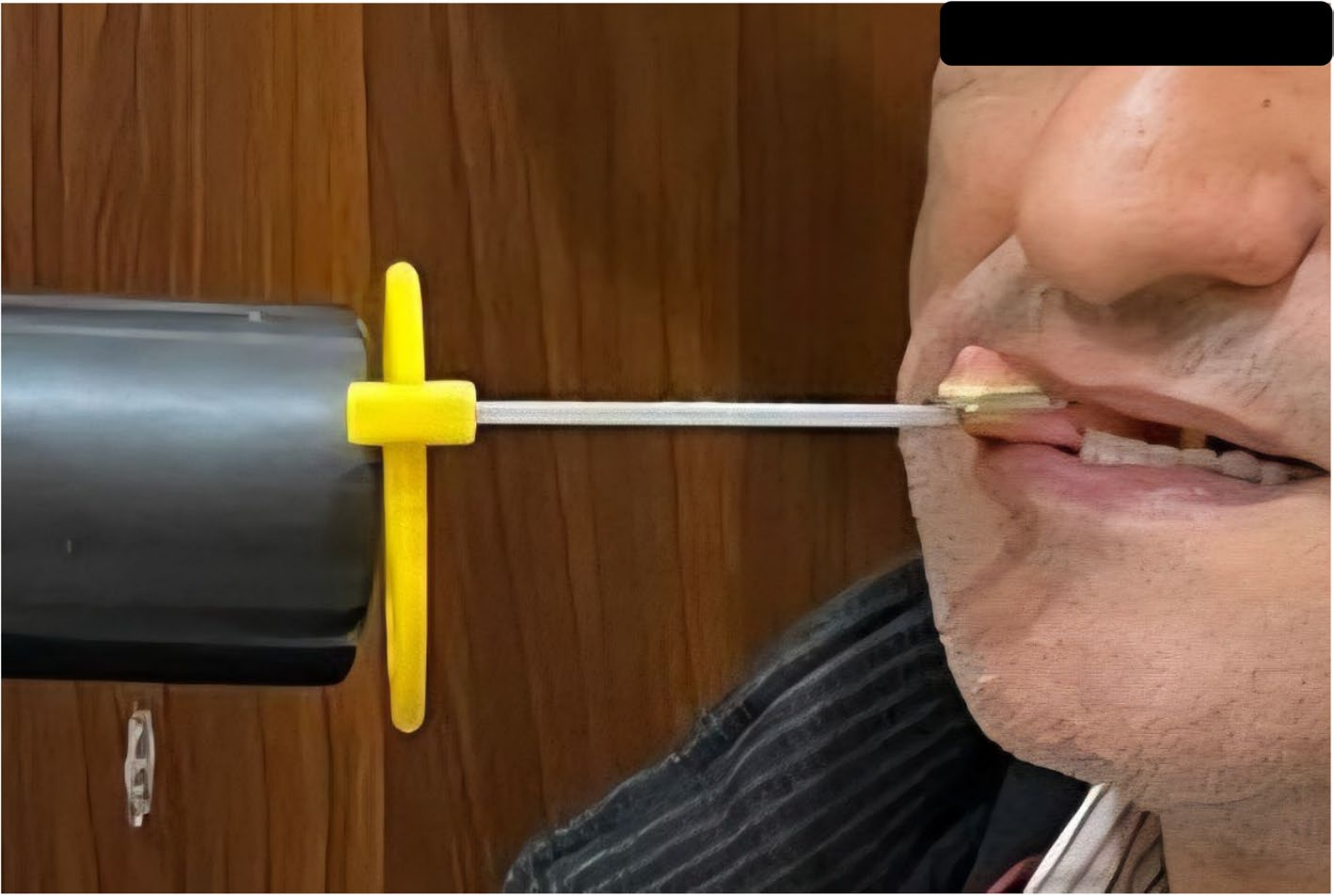
The film holder attached to the acrylic jig to obtain periapical X-rays

**Fig. 5:**
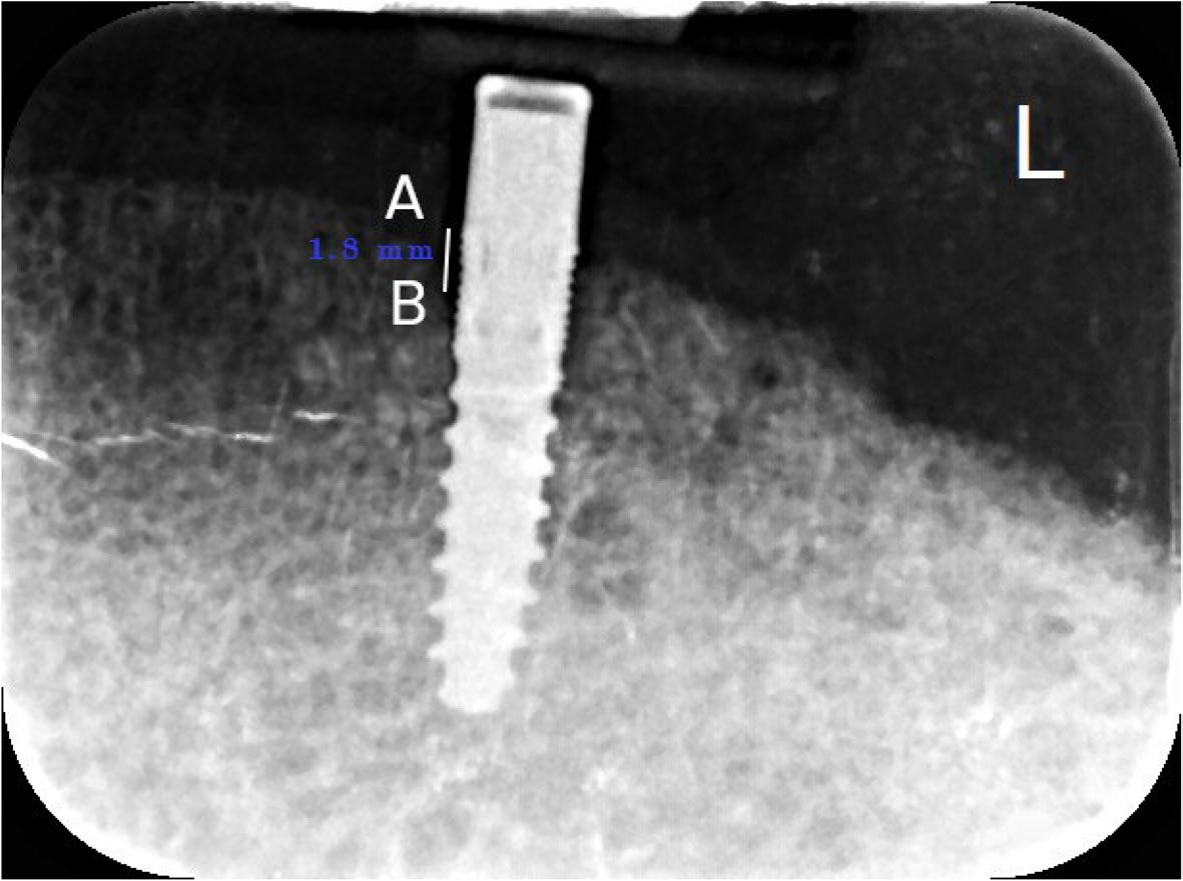
Periapical radiograph of implants. **A**: represents the implant shoulder, **B**: represents the initial bone-to-implant contact

A long cone paralleling technique was performed. As suggested by Elsyad et al. [41–43], to ensure standardization of the angle and distance between the X-ray sensor and the cone during the intraoral radiographs, an acrylic bite jig was constructed and attached to the film holder with auto-polymerized acrylic resin. The holder was used during subsequent film exposures to maintain a fixed position for each radiograph (Fig. 4). The pictures were taken with a digital X-ray sensor (Ai-dental, Woodpecker, China) and the dental X-ray machine (Acteon, de Gotzen^®^ S.r.l., Roma, Italy). To assess alveolar bone loss, the vertical bone level was measured by determining the distance in millimeters between the shoulder of the implant (A point) and the initial bone-to-implant contact (B point) on the AB line (Fig. 5) [43]. The amount of bone loss was determined by comparing radiographs taken at the time of overdenture placement (T0) and at one(T12), two(T24), and three(T36) years after insertion, measuring the vertical bone loss. To accurately measure peri-implant bone levels in the radiographs and to address distortion in the periapical film, we compared implant dimensions in the radiographs with actual implant dimensions. Alveolar bone loss was measured at the mesial and distal surfaces of each implant, and the average was subjected to statistical analysis.

Three independent calibrated examiners performed clinical (plaque index, gingival index, probing depth) and radiographic evaluations (marginal bone loss) after instruction and calibration by a periodontal staff member. The inter-examiner variability was tested using α (Cronbach’s) test. The measurements were considered reliable if the intra-class correlation coefficient was > 0.80.

## Retention measurement

**Fig. 6:**
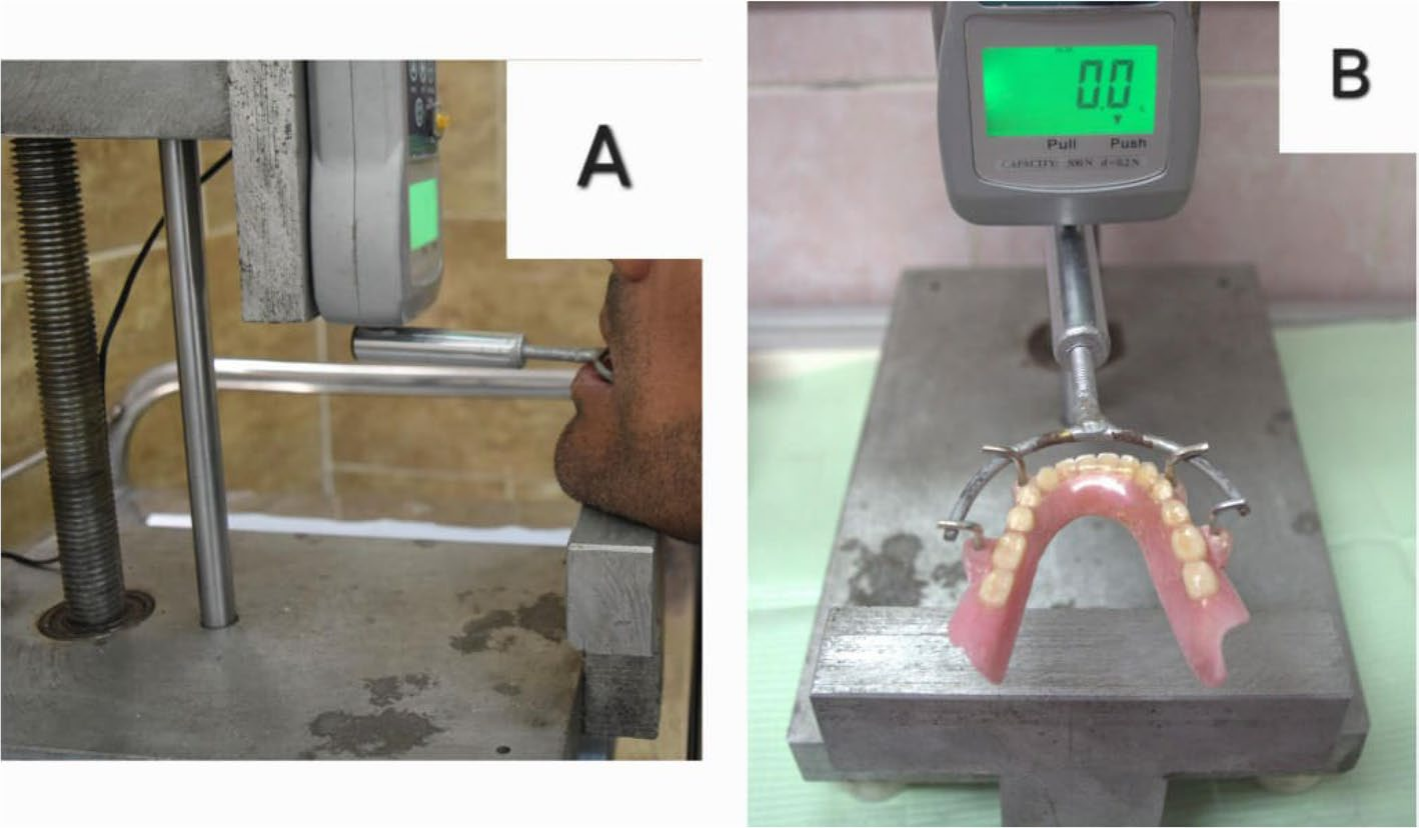
**A** The patient resting his chin on the chin support of the retention device. **B** Four hooks attached to the buccal flange perpendicular to the occlusal plane

Retention of implant overdenture prosthesis was measured for all groups on the day of insertion (T0), after 6 months (T6), and after 12 months (T12). The retention was measured using a digital force meter with a pure vertical pulling force that represents retention values in Newton [44]Four hooks were attached to the buccal flange at the canine and first molar position, perpendicular to the occlusal plane. The prosthesis was then placed in the patient’s mouth carefully to ensure full seating of the prosthesis. The patient placed his chin on the chin support of the retention device, and hooks were engaged with the device’s fork that was oriented parallel to the occlusal plane (Fig. 6). Pulling force was then applied to pull the prosthesis out of its place while the force meter registered the peak value. Data were independently recorded by 3 prosthodontic coinvestigators, and the mean was averaged and subjected to statistical analysis. The instruction, training, and calibration of investigators were performed in another study [44].

### Patient satisfaction (VAS)

A visual analog scale (VAS)_based questionnaire was used to assess patient satisfaction [32, 45]. Patients were instructed to indicate their level of satisfaction by marking their response on a 100-mm line, where zero denotes total dissatisfaction and 100 represents total satisfaction. For each question, a statistical analysis was performed on the mean of the responses (the length of the lines from zero to the marks in mm). The VAS questionnaire addresses several patient satisfaction factors not covered by OHRQoL domains [46]. The final version of the VAS questionnaire was translated into Arabic with the assistance of linguists collaborating with the writers. A convenient sample of twenty individuals who wear overdentures was selected to test the final Arabic version.

Participants were questioned to ensure comprehension of each questionnaire item and selected response. Consequently, the final Arabic version of VAS was updated and utilized in this investigation. The study population received the questionnaire from a single, blind dental staff member at the prosthodontic department. Participants independently completed the questionnaire once, without repeating any questions. The VAS questionnaires were measured 6 months after wearing their prosthesis.

### Statistical analysis

Baseline criteria were compared between groups using one-way ANOVA for continuous variables and the Chi-square test for categorical variables. A comparison of gingival and plaque scores between observations was made using the Friedman test, followed by the Wilcoxon signed-rank post hoc test. Between-group comparison of gingival and plaque scores was made using the Kruskal-Wallis test, followed by the Mann-Whitney post hoc test. Comparison of pocket depth, crestal bone loss, and retention values between observations and groups was made using a Linear mixed Model, followed by a Bonferroni post hoc test for multiple comparisons. Comparison of VAS questions between groups was made using one-way ANOVA, followed by a Bonferroni post hoc test. P-value < 0.05 was considered significant. The SPSS statistical package for the social sciences, version 25 (SPSS Inc., Chicago, IL, USA), was used for data analysis.

## Results

**Fig. 7:**
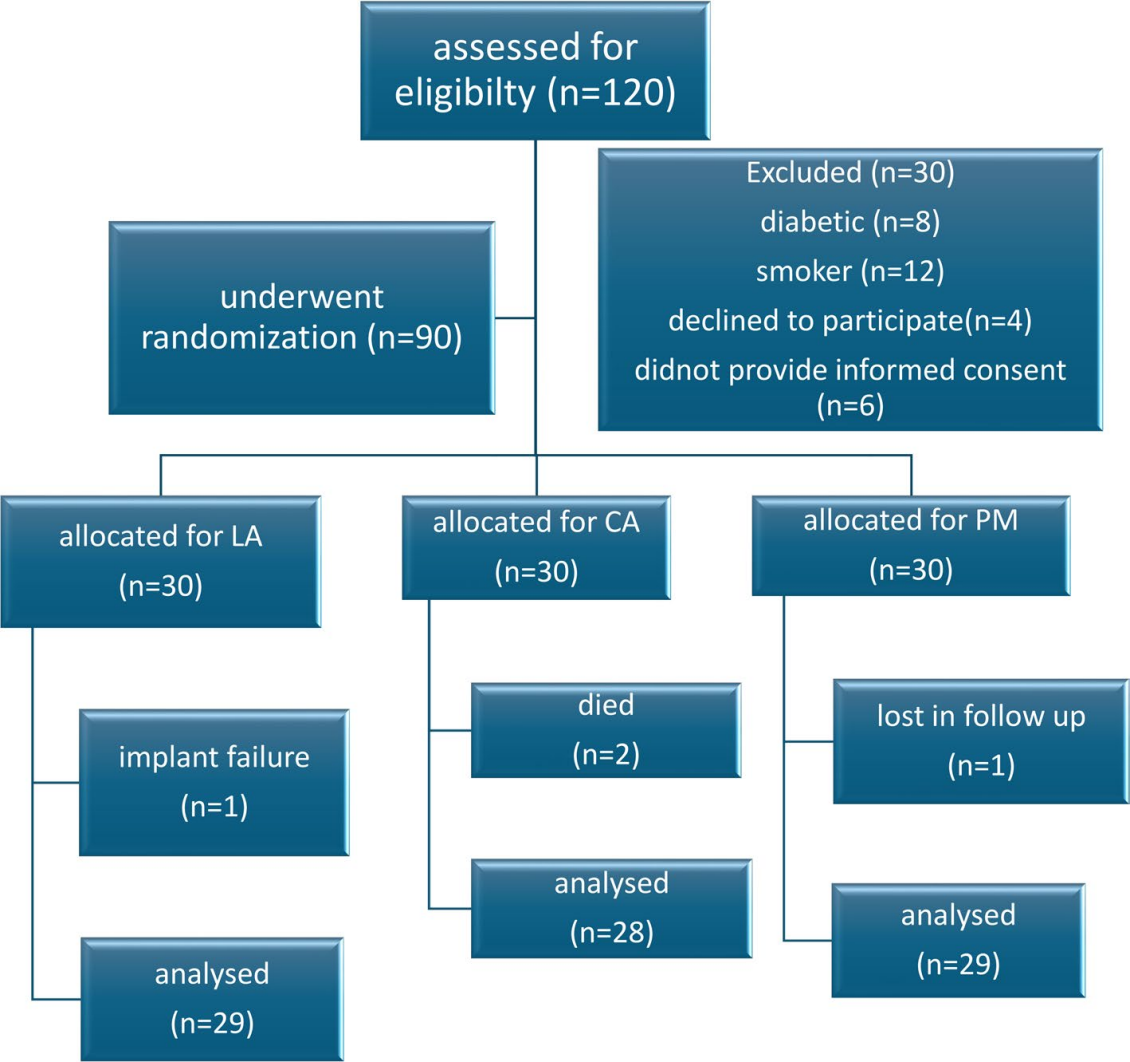
Patient flow diagram

A total of ninety patients were initially enrolled and randomized into three study groups. The flow of participants throughout all stages of the trial is outlined in Fig. 7. During the follow-up period, four patients were excluded from the final analysis. Specifically, one patient in Group LA experienced an early implant failure at 5 weeks and subsequently withdrew from the study; two patients in Group CA passed away 7 and 11 months post-placement; and one patient in Group PM was unable to complete the study due to serious medical reasons. As a result, the final analysis included 86 patients: 29 in Group LA, 28 in Group CA, and 29 in Group PM. While this final sample size was slightly lower than the initial target, it remained consistent with the study’s power calculation, which had anticipated some attrition.

Inter-examiner agreement for bone loss and retention values was assessed using the alpha chrombach test with an intraclass correlation coefficient > 0.85, indicating a good agreement. For all groups, plaque and gingival scores increased significantly with time. (Table 2). Plaque and gingival scores did not significantly differ across groups at T0. At T6 and T12, the highest plaque and gingival scores were noted with the premolar group, followed by the lateral group, and the lowest scores were observed with the canine group. There was no significant difference in plaque and gingival scores between the lateral and premolar groups. Both lateral and premolar groups showed significantly higher plaque and gingival scores than the canine groups.

**Table 2:** Comparison of plaque and gingival scores between groups and observation times

	T0 M (min-max)	T6 M (min-max)	T12 M (min-max)	Effect size	Freidman *P* value
Plaque index					
* Lateral group*	0.00 Aa (0.00–1.00)	0.50 ABb (0.00–1.00)	1.00 Ac (1.00–2.00)	0.659	< 0.001*
* Canine group*	0.00 Aa (0.00–1.00)	0.00 Bab (0.00–1.00)	0.00 Bb (0.00–2.00)	0.168	< 0.001*
* Premolar group*	0.00 Aa (0.00–1.00)	1.00 Ab (0.00–2.00)	1.00 Ac (0.00–2.00)	0.465	< 0.001*
*Effect size*	0.044	0.119	0.301		
*Kruskal-Wallis test* *P value*	0.123	0.004*	< 0.001*		
Gingival index					
* Lateral group*	0.00 Aa (0.00–1.00)	0.50 Ab (0.00–1.00)	1.00 Ac (1.00–2.00)	0.607	< 0.001*
* Canine group*	0.00 Aa (0.00–1.00)	0.00 Bab (0.00–1.00)	0.00 Bb (0.00–2.00)	0.111	0.001*
* Premolar group*	0.00 Aa (0.00–1.00)	1.00 Aab (0.00–1.00)	1.00 Ab (0.00–2.00)	0.288	< 0.001*
*Effect size*	0.045	0.146	0.300		
*Kruskal Wallis test* *P value*	0.122	0.002*	< 0.001*		

*M* Median, *min* minimum, *max* maximum

At 5%, *P is significant. A significant difference between groups was indicated by different uppercase letters in the same column (Mann-Whitney test, *p*<.05). A significant difference between observation times is shown by different lowercase letters in the same row (Wilcoxon signed-rank test, *p*<.05)

For the lateral group, there was no significant difference in pocket depth between observation times. Pocket depth increased significantly with time for canine and premolar groups (Table 3). For all observations, there was a significant difference in pocket depth between groups. For all observations, the highest pocket depth was noted with the premolar group, followed by the canine group, and the lowest pocket depth was noted with the lateral group. At T0, there was no significant difference in pocket depth between the canine and premolar groups. At T6 and T12, there was a significant difference in pocket depth between the 2 groups.

**Table 3:** Comparison of Pocket depth between groups and observation times

	T0 X ± SD	T6 X ± SD	T12 X ± SD	Effect size	Repeated measures ANOVA *P* value
Pocket depth					
* Lateral group*	0.19±.06Aa	0.25±.03Aa	0.27±.03Aa	0.138	0.551
* Canine group*	0.94±.54Ba	1.09±.51Bab	1.22±.50Bb	0.525	0.049*
* Premolar group*	0.97±.06Ba	1.40±.06Cb	1.90±.06Cc	0.941	< 0.001*
Effect size	0.575	0.962	0.877		
Repeated measures ANOVA P value	0.021*	0.005*	< 0.001*		

^*^P at 5% significance level; X; mean, SD standard deviation. A significant difference between groups was indicated by different uppercase letters in the same column (Bonferroni test, *p*<.05). A significant difference between observation periods is shown by different lowercase letters in the same raw (Bonferroni test, *p*<.05)

For all groups, crestal bone loss increased significantly with time (Table 4). All observations showed a significant difference in crestal bone loss between groups. For all observations, the highest crestal bone loss was noted with the premolar group, followed by the canine group, and the lowest bone loss was noted with the lateral group. In all observations, there was no significant difference in bone loss between canine and premolar groups. Canine and premolar groups recorded significantly higher bone loss than the lateral group.

**Table 4:** Comparison of marginal bone loss between groups and observation times

	T12 X ± SD	T24 X ± SD	T36 X ± SD	Effect size	Repeated measures ANOVA *P* value
Crestal bone loss					
* Lateral group*	0.64±.09Aa	0.46±.15Ab	0.18 ± 0.21Ac	0.849	0.002*
* Canine group*	1.14±.25Ba	1.44 ± 0.29Bb	1.65 ± 0.29Bc	0.846	< 0.001*
* Premolar group*	1.27±.12Ba	1.60 ± 0.17Bb	1.99 ± 0.14Bc	0.623	< 0.001*
Effect size	0.781	0.880	0.944		
Repeated measures ANOVA P value	0.001*	< 0.001*	< 0.001*		

^*^P is significant at 5%; X; mean, SD standard deviation. A significant difference between the groups was indicated by different uppercase letters in the same column (Bonferroni test, *p*<.05). A significant difference in observation times is shown by different lowercase letters in the same raw (Bonferroni test, *p*<.05)

All groups’ retention values decreased significantly with time (Table 5). All observations showed a significant difference in retention values between groups. For all observations, the highest retention forces were noted with the premolar group, followed by the canine group, and the lowest retention values were noted with the lateral group. In all observations, there was a significant difference in retention values between the 2 groups.

**Table 5:** Comparison of retention between groups and observation times

	T0 X ± SD	T6 X ± SD	T12 X ± SD	Effect size	Repeated measures ANOVA *P* value
Retention					
* Lateral group*	25.34 ± 1.59Aa	17.72 ± 1.14Ab	13.16 ± 1.18Ac	0.892	< 0.001*
* Canine group*	33.26 ± 1.12Ba	24.66 ± 3.38Bb	20.48±.75Bc	0.903	< 0.001*
* Premolar group*	41.60 ± 2.25Ca	31.40 ± 2.60Cb	23.29 ± 3.73Cc	0.944	< 0.001*
Effect size	0.949	0.857	0.826		
Repeated measures ANOVA P value	< 0.001*	< 0.001*	< 0.001*		

^*^P at 5% significance level; X; mean, SD standard deviation. A significant difference between groups was indicated by different uppercase letters in the same column (Bonferroni test, *p*<.05). A significant difference between observation periods is shown by different lowercase letters in the same raw (Bonferroni test, *p*<.05)

The results of the patient satisfaction questionnaire are presented in Table 6. Significant differences were observed between groups for all VAS questions, except for ease of cleaning. The satisfaction scores of the CD group were lower than other groups for satisfaction with the mandibular prosthesis, comfort, ease of speaking, handling, and embarrassment. However, there were no significant differences in satisfaction scores between the LA, CA, and PM. In terms of retention, stability, and occlusion of the mandibular prosthesis, the highest patient satisfaction was noted in the premolar group, followed by the canine group (with no difference), then the lateral group, with the lowest satisfaction scores observed in the CD group.

**Table 6:** Results of patient satisfaction between groups

	CD		Lateral		Canine		Premolar		Effect size	ANOVA *P* value
	X	SD	X	SD	X	SD	X	SD		
Mandibular prosthesis	41.50a	7.84	80.00b	7.45	78.00b	9.19	79.44b	7.26	0.825	< 0.001*
Retention of the prosthesis	34.50a	4.38	69.50b	5.50	74.00c	3.94	76.11c	4.17	0.940	< 0.001*
Stability of the prosthesis	28.00a	4.83	62.50b	5.89	68.50c	5.30	69.44c	5.83	0.916	< 0.001*
Occlusion of the prosthesis	28.00a	5.37	71.00b	5.68	76.00c	3.94	76.11c	5.46	0.954	< 0.001*
Comfort with prosthesis	34.50a	6.85	75.00b	7.45	78.50b	4.74	76.67b	6.61	0.900	< 0.001*
Ease of cleaning of the prosthesis	82.00a	7.53	78.00a	5.37	80.00a	5.27	80.56a	5.27	0.062	0.518
Ease of speaking	33.00a	7.89	82.50b	7.91	79.50b	7.25	82.78b	7.12	0.898	< 0.001*
Ease of chewing	27.00a	6.32	78.50b	7.47	83.50b, c	6.69	87.78c	7.12	0.934	< 0.001*
Handling of a prosthesis	80.50a	7.62	66.50b	5.80	71.50b	6.69	71.11b	7.41	0.381	0.001*
Embarrassing	85.50a	7.62	35.00b	7.45	33.50b	7.47	31.11b	6.51	0.916	< 0.001*

^*^p is significant at 5% for X, mean, SD, and standard deviation. Group differences are significant when different letters appear in the same raw (Bonferroni post hoc test, *p*<.05). According to the Bonferroni post hoc test, a non-significant difference between groups is shown by similar letters in the same raw (*p*>.05)

The premolar and canine groups exhibited significantly higher satisfaction levels compared to the lateral group. The highest satisfaction with the ease of chewing was noted in the premolar group, followed by the canine group (with no discernible difference between the two), then the lateral group, and the lowest satisfaction scores were observed in the CD group. There were no significant differences in satisfaction scores between the lateral and canine groups or between the canine and premolar groups. However, there was a difference between the lateral and premolar groups.

## Discussion

The findings regarding peri-implant soft tissue changes indicate a significant increase in plaque and gingival scores over time for all groups. Consistent with this, ELsyad et al. [47] observed a significant rise in these scores over time with different implant positions for two-implant overdentures. Higher food stagnation and plaque accumulation near the Locator attachment’s abutment, as well as poor oral hygiene, possibly brought on by the patients’ decreased hand dexterity, may be linked to the increased gingival and plaque indices. On the contrary, other studies [18, 48] have reported that these parameters did not show significant changes over time when using Locator attachments, possibly because participants in those studies followed strict oral hygiene practices. Both lateral and premolar groups showed significantly higher plaque and gingival scores than canine groups. For the LA group, the increased gingival and plaque scores can be attributed to the small inter-implant distance that could interfere with the proper cleansing [49, 50]. while the high plaque and gingival scores in the PM group could be attributed to the more posterior position of the implants, which can be more inaccessible for the patient to achieve proper measures of oral hygiene [51]. The highest pocket depth was noted in the premolar group, and the lowest pocket depth was observed in the lateral group. This could be explained by the increase in peri-implant bone loss in the premolar group, as shown in the results of this study. Similar results were found in a previous clinical study [47], in which the authors reported higher crestal bone loss and pocket depth in the premolar region.

The highest crestal bone loss was noted in the premolar group, and the lowest bone loss was observed in the lateral group. This could be attributed to higher stresses in the premolar region compared to a more anterior position of the implants. In agreement with these findings, Patil et al. [21] and Hong et al. [23] in their in vitro study found that more posterior positioning of the implants exhibited higher stress concentration and the lowest stresses when implants were placed in the lateral incisor region for two-implant overdentures. From a biomechanical perspective, the greater marginal bone loss in the premolar area, compared to the canine and lateral incisor areas, may be attributed to many reasons. When over-dentures are supported by two implants, they tend to pivot around a fulcrum line that connects the two implants [23]. A larger “seesaw effect” and higher stresses on the implants in the premolar position result from the fulcrum line being positioned more posteriorly. In contrast, implants placed more anteriorly cause the fulcrum line to shift forward, reducing the “seesaw effect " [23]. 

The proximity of implants to the first molar region, where edentulous patients commonly apply the greatest occlusal stresses due to the contraction of the elevator muscles, may be the cause of the higher bone loss associated with implants in the premolar (PM) group [52]. Furthermore, due to mandibular anatomy found on the lingual side of the mandible, most implants placed in premolar regions are inclined lingually [53]. This tilt may increase peri-implant bone loss and dramatically raise peri-implant stresses, particularly when Locator attachments are used [54–56]. The lower bone loss values observed in the LA group may be attributed to the more anterior position of the implants supporting the overdenture, which reduces rotational movement during anterior vertical incising forces. Conversely, during posterior loading, the overdenture experiences minimal rotation due to the larger denture-bearing area in the molar region, which provides stronger resistance against occlusal forces [22]. In accordance with this elucidation, a study by Hong et al. [23], revealed that overdentures retained by implants placed in the lateral incisor area exhibited reduced peri-implant bone stresses in comparison to implants placed in the premolar and canine areas when force was applied to the first molars. However, the interpretation of increased marginal bone loss at the premolar position and reduced marginal bone loss at the lateral incisor position should be approached with great caution due to the substantial drop-out rate and the resulting failure to meet the predefined statistical power. Therefore, to draw firm conclusions, further validation is required in adequately powered studies.

The observed reduction in final retention values of locator attachments at twelve months, as compared to the initial values, aligns with previous in vitro studies and is not unexpected [57–61]. This decrease in retention can be attributed to surface alterations, wear, and tear of the nylon inserts [62]. Regarding retention values, the premolar location consistently exhibited higher retention values compared to the canine and incisor locations at all measuring times. This could be attributed to the increased inter-implant distance at the premolar position, which positively affects attachment retention values. Furthermore, the lingual inclination of implants inserted at the premolar location enhances retention values by creating undercuts in the locator inserts between internal and external frictional flanges. In agreement with this explanation, multiple in vitro studies have documented an increase in resilient attachment retention with heightened implant angulation [61, 63]. In line with the findings of Scherer D M et al., [24] observed an increase in vertical retention values with distal implants up to the second premolar position, with lower retention values observed for the incisor position.

Patients showed greater satisfaction with implant overdentures, regardless of implant position, compared to conventional complete dentures (CD). This could be attributed to the attachments, which significantly influenced the retention and stability of overdentures and consequently enhanced patient satisfaction. Additionally, patients with atrophied mandibular ridges may experience denture instability due to increased muscular attachment, which may explain their decreased satisfaction with complete dentures [64]. 

Patients reported higher satisfaction with the retention, stability, and occlusion at the premolar (PM) position compared to the canine (CA) and lateral anterior (LA) positions. These findings are consistent with the higher retention values at the PM location as opposed to the CA and LA locations. The degree of stability and retention of the dentures, facilitated by the attachment method, significantly influences the patient’s level of satisfaction [33]. The presence of attachments at the PM location, which represents the patient’s preferred chewing area, could be associated with high satisfaction regarding ease of chewing.

The clinical research findings may be limited by the small number of participants and patient dropouts. Although digital forcemeters can provide precise and standardized measurements of implant overdenture retention, several limitations should be noted, including potential challenges related to force direction, reproducibility, and wear simulation. Future clinical studies with large patient samples and extended follow-up periods are needed to confirm the findings of this study and to evaluate prosthetic complications and maintenance.

## Conclusion

The lateral incisor position for two-implant mandibular overdentures could preserve peri-implant bone, while the canine position may improve peri-implant soft tissue health. The premolar position may enhance retention and patient satisfaction with overdentures. Consequently, the three implant positions may be used successfully, as they did not affect the implant success. However, future studies are needed to evaluate the long-term prosthetic complications and maintenance of the three implant positions before recommending a specific implant position.

## Data Availability

The datasets used in the current study are available from the corresponding author upon request.
